# A Rare Complication of Abdominal Aortic Aneurysm: A Case Report of Aortocaval Fistula

**DOI:** 10.7759/cureus.38106

**Published:** 2023-04-25

**Authors:** Raghad S Aldahman, Saleh A Alghadouni, Ahlam S Alharbi

**Affiliations:** 1 General Practice, Qassim University, Qassim, SAU; 2 Family Medicine, Primary Health Care Center, Riyadh, SAU

**Keywords:** case report, hemodynamic instability, back pain, inferior vena cava, abdominal aortic aneurysm, aortocaval fistula

## Abstract

Aortocaval fistula is a rare but serious complication of an abdominal aortic aneurysm characterized by communication between the dilated abdominal aorta and inferior vena cava. Prompt diagnosis and treatment are essential to reduce the mortality rate. A 66-year-old man with a history of poorly controlled hypertension, diabetes mellitus, and dyslipidemia presented to the emergency department with sudden, severe lower back pain. Laboratory investigations showed a rapid drop in hemoglobin levels and increased lactate levels. A CT scan revealed an aortocaval fistula resulting from a rupture of the abdominal aorta. The patient underwent emergency surgery, but a cardiac arrest occurred during the procedure, and he could not be resuscitated. Despite advances in imaging and surgical techniques, the mortality rate of aortocaval fistula remains high. It is crucial for clinicians to maintain a high level of suspicion for aortocaval fistula in patients with an abdominal aortic aneurysm who present with a sudden onset of abdominal and back pain and to initiate appropriate resuscitative measures and an urgent surgical consultation.

## Introduction

An aortocaval fistula is a serious and rare complication of abdominal aortic aneurysm, which is characterized by localized dilatation of the abdominal aorta with a diameter greater than 3.0 cm [1]. Although aortocaval fistula is a relatively uncommon complication, it can be life-threatening and requires prompt diagnosis and treatment [2]. In this condition, the aneurysm erodes into the inferior vena cava, leading to communication between the two vessels. The exact prevalence of aortocaval fistula is unknown, but estimates suggest that it occurs in only a small percentage (0.04% to 0.07%) of all abdominal aortic aneurysms [1,2]. However, men over the age of 65 years with a history of chronic abdominal aortic aneurysms are at higher risk of developing this complication [1,2].

The symptoms of aortocaval fistula can vary, and the most common symptom is a pulsatile abdominal mass. The severity and duration of symptoms depend on the size, location, and extent of the fistula [2]. It is crucial to diagnose and treat aortocaval fistula promptly since untreated aortocaval fistula has a mortality rate that approaches 100% [1]. In this case, we present a rare and fatal case of aortocaval fistula in a patient who initially presented with low back pain for three weeks.

## Case presentation

A 66-year-old retired man presented to the emergency department with sudden severe back pain primarily located in his lower back. He described the pain as a constant, dull ache with occasional sharp, shooting pains, which were exacerbated by movement, particularly bending and twisting. The pain had gradually worsened over the course of three weeks and had become severe in the hours leading up to the patient's presentation to the emergency department. Over-the-counter pain medication did not alleviate the pain.

Upon further investigation into the patient's medical history, it was discovered that the patient had a 20-year history of poorly controlled hypertension, diabetes mellitus, or dyslipidemia. Regarding social history, the patient was retired and lived alone. He denied any tobacco or alcohol use. The patient reported a sedentary lifestyle, with little physical activity beyond basic activities of daily living. There was no significant family history of cardiovascular or vascular disease.

On examination, the patient was found to be hypotensive with a blood pressure of 80/40 mmHg, tachycardic with a heart rate of 120 beats per minute, and tachypneic with a respiratory rate of 28 breaths per minute. Other vital signs were within normal limits.

Laboratory investigations revealed a drop in hemoglobin levels from 13.5 g/dL to 10.5 g/dL within two hours of presentation, indicating significant bleeding. The patient also had an elevated lactate level of 6.2 mmol/L, which suggested poor tissue perfusion, with a markedly elevated D-dimer level of >20 µg/mL. Arterial blood gas analysis showed acidosis, with a pH of 7.25 and decreased bicarbonate levels of 16 mEq/L. These findings are consistent with tissue hypoxia and organ dysfunction resulting from significant blood loss and poor tissue perfusion (Table 1).

**Table 1:** Laboratory investigations

Lab Test	Result	Reference Range
Hemoglobin	13.5 g/dL	13.5-17.5 g/dL
Hemoglobin (two hours)	10.5 g/dL	13.5-17.5 g/dL
White blood cell count	12,000/mm³	4,500-11,000/mm³
Platelet count	200,000/mm³	150,000-450,000/mm³
Serum creatinine	1.2 mg/dL	0.7-1.3 mg/dL
Blood urea nitrogen	20 mg/dL	7-20 mg/dL
Troponin T	0.1 ng/mL	<0.1 ng/mL
D-dimer	>20 µg/mL	<0.5 µg/mL
Lactate	6.2 mmol/L	0.5-1.6 mmol/L
Arterial pH	7.25	7.35-7.45
Bicarbonate	16 mEq/L	22-28 mEq/L
PaO_2_	60 mmHg	75-100 mmHg
PaCO_2_	38 mmHg	35-45 mmHg

**Figure 1:**
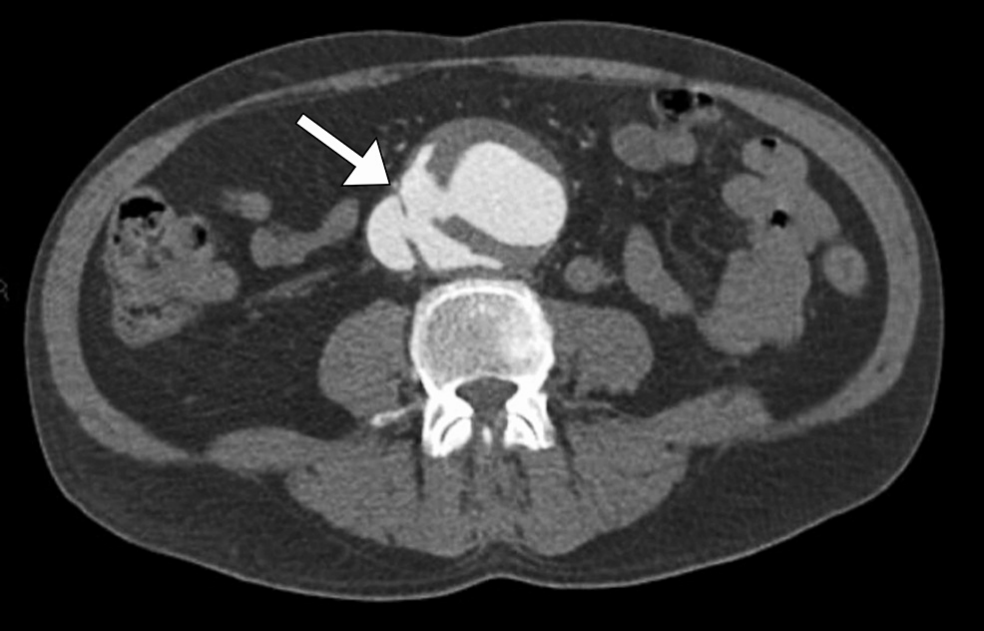
Axial CT image demonstrating an aneurysmal dilatation of the infrarenal abdominal aorta and an abnormal connection between the aneurysm and the inferior vena cava (arrow)

Further investigation was performed with a CT scan of the abdomen and pelvis, which revealed aneurysmal dilatation of the infra-renal abdominal aorta with aortocaval fistulous communication. This revealed the diagnosis of an aortocaval fistula and indicated a rupture of the posterior wall of the abdominal aorta with a fistulous communication between the aorta and the inferior vena cava (Figure 1).

The patient was immediately taken for emergency surgery, where aortocaval fistula surgical repair was attempted. However, the patient suffered a cardiac arrest during the procedure and the resuscitation was not successful.

## Discussion

Aortocaval fistula is a rare complication of abdominal aortic aneurysm that can cause rapid hemodynamic instability and is associated with a high mortality rate. The fistula forms between the dilated abdominal aorta and the inferior vena cava, resulting in a shunt of blood from the high-pressure arterial system to the low-pressure venous system. This can lead to decreased arterial perfusion and oxygen delivery to the vital organs, resulting in shock and organ dysfunction [1].

The clinical presentation of the aortocaval fistula is typically the sudden onset of abdominal and back pain, associated with signs and symptoms of hypovolemia and shock, including tachycardia, hypotension, and diaphoresis. The diagnosis is usually confirmed with imaging studies, such as CT angiography or ultrasound, which can demonstrate the presence of an abdominal aortic aneurysm and a fistulous communication with the inferior vena cava [3].

Treatment of an aortocaval fistula is an emergent surgical intervention, which involves repair of the aneurysm and closure of the fistula [1,4]. The surgery can be technically challenging and carries a high risk of mortality and morbidity. In some cases, endovascular repair may be considered an alternative to open surgery, but this is typically reserved for patients who are not surgical candidates [3-5].

The prognosis of the aortocaval fistula is poor. The high mortality is attributed to the rapid onset of hemodynamic instability, significant blood loss, and organ dysfunction that often occurs before diagnosis and surgical intervention [1]. In addition, patients with abdominal aortic aneurysms at risk of developing aortocaval fistula are often elderly and have multiple comorbidities, which further increases the risk of adverse outcomes [2].

There are several risk factors associated with the development of aortocaval fistula, including large abdominal aortic aneurysm size, rapid aneurysm growth, and the presence of a mural thrombus [1]. Other reported risk factors include female gender, history of prior abdominal aortic aneurysm repair, and use of anticoagulant or antiplatelet therapy [1,2]. However, the exact mechanisms by which these risk factors contribute to developing aortocaval fistula are poorly understood [2,3].

## Conclusions

Aortocaval fistula is a rare yet potentially life-threatening complication of abdominal aortic aneurysm that requires prompt identification and immediate surgical intervention. When patients with suspected abdominal aortic aneurysm present with sudden onset of abdominal and back pain accompanied by hemodynamic instability, healthcare providers should consider aortocaval fistula as a possible differential diagnosis. Maintaining a high degree of suspicion is critical, and appropriate resuscitative measures should be initiated while an urgent surgical evaluation is arranged. Prompt recognition and management of aortocaval fistula are crucial for improving patient outcomes and reducing mortality rates.

## References

[1] Patelis N, Giagkos GC, Maltezos K, Klonaris C (2018). Aortocaval fistula: an unusual complication of ruptured abdominal aortic aneurysm. BMJ Case Rep.

[2] Greenfield S, Martin G, Malina M, Theivacumar NS (2020). Aortocaval fistula, a potentially favourable complication of abdominal aortic aneurysm rupture in endovascular repair. Ann R Coll Surg Engl.

[3] Esmat HA, Naseri MW (2021). Endovascular management of aortocaval fistula complicating abdominal aortic aneurysm presenting as an acute renal failure. Ann Med Surg (Lond).

[4] Orion KC, Beaulieu RJ, Black JH 3rd (2016). Aortocaval fistula: is endovascular repair the preferred solution?. Ann Vasc Surg.

[5] van de Luijtgaarden KM, Bastos Gonçalves F, Rouwet EV, Hendriks JM, Ten Raa S, Verhagen HJ (2013). Conservative management of persistent aortocaval fistula after endovascular aortic repair. J Vasc Surg.

